# Patterns and predictors of multiple sclerosis phenotype transition

**DOI:** 10.1093/braincomms/fcae422

**Published:** 2024-11-23

**Authors:** Luigi Pontieri, Nupur Greene, Malthe Faurschou Wandall-Holm, Svend Sparre Geertsen, Nasrin Asgari, Henrik Boye Jensen, Zsolt Illes, Jakob Schäfer, Rikke Marie Jensen, Tobias Sejbæk, Arkadiusz Weglewski, Mie Reith Mahler, Mai Bang Poulsen, Sivagini Prakash, Morten Stilund, Matthias Kant, Peter Vestergaard Rasmussen, Kristina Bacher Svendsen, Finn Sellebjerg, Melinda Magyari

**Affiliations:** The Danish Multiple Sclerosis Registry, Department of Neurology, Copenhagen University Hospital—Rigshospitalet Glostrup, 2600 Glostrup, Denmark; Sanofi, Cambridge, MA 02141, USA; The Danish Multiple Sclerosis Registry, Department of Neurology, Copenhagen University Hospital—Rigshospitalet Glostrup, 2600 Glostrup, Denmark; Sanofi, Cambridge, MA 02141, USA; Department of Neurology, Naestved, Slagelse and Ringsted Hospitals, 4200 Slagelse, Denmark; Department of Regional Health Research, University of Southern Denmark, 5000 Odense, Denmark; Department of Molecular Medicine, University of Southern Denmark, 5000 Odense, Denmark; Department of Regional Health Research, University of Southern Denmark, 5000 Odense, Denmark; Department of Brain and Nerve Diseases, Lillebælt Hospital, 6000 Kolding, Denmark; Department of Neurology, Odense University Hospital, 5000 Odense, Denmark; Department of Clinical Research, University of Southern Denmark, 5000 Odense, Denmark; Department of Neurology, Aalborg University Hospital, 9100 Aalborg, Denmark; Danish Multiple Sclerosis Center, Department of Neurology, Copenhagen University Hospital—Rigshospitalet Glostrup, 2600 Glostrup, Denmark; Department of Regional Health Research, University of Southern Denmark, 5000 Odense, Denmark; Department of Neurology, Southwest Jutland Hospital, University Hospital of Southern Denmark, 6700 Esbjerg, Denmark; Department of Neurology, Herlev and Gentofte Hospital, 2730 Herlev, Denmark; Department of Clinical Medicine, Faculty of Health and Medical Sciences, University of Copenhagen, 2200 Copenhagen, Denmark; The Danish Multiple Sclerosis Registry, Department of Neurology, Copenhagen University Hospital—Rigshospitalet Glostrup, 2600 Glostrup, Denmark; Danish Multiple Sclerosis Center, Department of Neurology, Copenhagen University Hospital—Rigshospitalet Glostrup, 2600 Glostrup, Denmark; Department of Neurology, Copenhagen University Hospital—North Zealand, 3400 Hillerød, Denmark; Department of Neurology, Viborg Regional Hospital, 8800 Viborg, Denmark; Department of Neurology, Physiotherapy and Occupational Therapy, Gødstrup Hospital, 7400 Herning, Denmark; NIDO | Centre for Research and Education, Gødstrup Hospital, 7400 Herning, Denmark; Department of Neurology, Southern Jutland Hospital, University of Southern Denmark, 6200 Aabenraa, Denmark; Department of Neurology, Aarhus University Hospital, 8200 Aarhus N, Denmark; Department of Neurology, Aarhus University Hospital, 8200 Aarhus N, Denmark; Danish Multiple Sclerosis Center, Department of Neurology, Copenhagen University Hospital—Rigshospitalet Glostrup, 2600 Glostrup, Denmark; Department of Clinical Medicine, Faculty of Health and Medical Sciences, University of Copenhagen, 2200 Copenhagen, Denmark; The Danish Multiple Sclerosis Registry, Department of Neurology, Copenhagen University Hospital—Rigshospitalet Glostrup, 2600 Glostrup, Denmark; Danish Multiple Sclerosis Center, Department of Neurology, Copenhagen University Hospital—Rigshospitalet Glostrup, 2600 Glostrup, Denmark; Department of Clinical Medicine, Faculty of Health and Medical Sciences, University of Copenhagen, 2200 Copenhagen, Denmark

**Keywords:** multiple sclerosis, secondary progressive multiple sclerosis, registry study, real-world data

## Abstract

Currently, there are limited therapeutic options for patients with non-active secondary progressive multiple sclerosis. Therefore, real-world studies have investigated differences between patients with relapsing-remitting multiple sclerosis, non-active secondary progressive multiple sclerosis and active secondary progressive multiple sclerosis. Here, we explore patterns and predictors of transitioning between these phenotypes. We performed a cohort study using data from The Danish Multiple Sclerosis Registry. We included patients with a relapsing-remitting phenotype, registered changes to secondary progressive multiple sclerosis and subsequent transitions between relapsing and non-relapsing secondary progressive multiple sclerosis, which was defined by the presence of relapses in the previous 2 years. We analysed predictors of transitioning from relapsing-remitting multiple sclerosis to relapsing and non-relapsing secondary progressive multiple sclerosis, as well as between the secondary progressive states using a multi-state Markov model. We included 4413 patients with relapsing-remitting multiple sclerosis. Within a median follow-up of 16.2 years, 962 were diagnosed with secondary progressive multiple sclerosis by their treating physician. Of these, we classified 729 as non-relapsing and 233 as relapsing secondary progressive multiple sclerosis. The risk of transitioning from relapsing-remitting to non-relapsing secondary progressive multiple sclerosis included older age (hazard ratio per increase of 1 year in age: 1.044, 95% confidence interval: 1.035–1.053), male sex (hazard ratio for female: 0.735, 95% confidence interval: 0.619–0.874), fewer relapses (hazard ratio per each additional relapse: 0.863, 95% confidence interval: 0.823–0.906), higher expanded disability status scale (hazard ratio per each additional point: 1.522, 95% confidence interval: 1.458–1.590) and longer time on disease-modifying therapies (hazard ratio per increase of 1 year in treatment, high-efficacy disease-modifying therapy: 1.095, 95% confidence interval: 1.051–1.141; hazard ratio, moderate-efficacy disease-modifying therapy: 1.073, 95% confidence interval: 1.051–1.095). We did not find significant predictors associated with the transition from relapsing secondary progressive multiple sclerosis to non-relapsing secondary progressive multiple sclerosis, whereas older age (hazard ratio per increase of 1 year in age: 0.956, 95% confidence interval: 0.942–0.971) prevented the transition from non-relapsing secondary progressive multiple sclerosis to relapsing secondary progressive multiple sclerosis. Our study suggests that transitioning from relapsing-remitting multiple sclerosis to non-relapsing secondary progressive multiple sclerosis depends on well-known factors affecting diagnosing secondary progressive multiple sclerosis. Further transitions between non-relapsing and relapsing secondary progressive multiple sclerosis are only affected by age. These findings add to the knowledge of non-active secondary progressive multiple sclerosis, a patient group with unmet needs in terms of therapies.

## Teaser

Pontieri *et al*. found that 22% of patients diagnosed with relapsing-remitting multiple sclerosis had transitioned to secondary progressive multiple sclerosis after 22 years, and the majority were no longer experiencing relapses. These data support the view of multiple sclerosis being a continuum with varying contributions of relapsing and progressive biology.

## Graphical Abstract

**Graphical Abstract:**
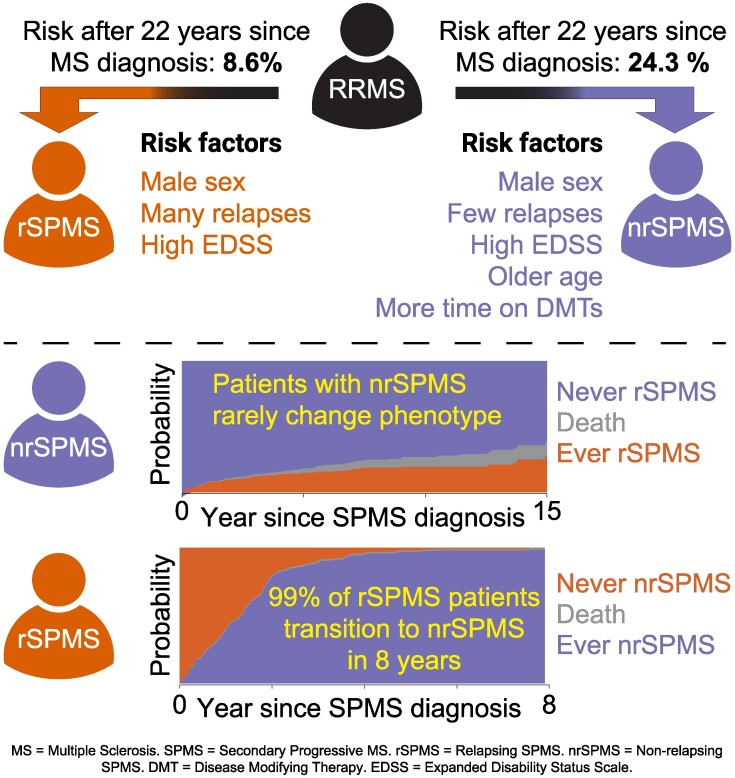

## Introduction

Multiple sclerosis is a chronic inflammatory, demyelinating disease of the CNS affecting 2.9 million people worldwide.^1^ Most people with multiple sclerosis (∼85%) are diagnosed with a relapsing-remitting phenotype (RRMS), characterized by relapses followed by periods of partial or complete recovery (remissions), whereas the remaining ∼15% are diagnosed with a primary progressive phenotype, characterized by a progressive accumulation of disability from the onset.^2,3^ Over time, a sizeable proportion of people with RRMS (up to 50% 30 years after onset)^4^ transition to secondary progressive multiple sclerosis (SPMS), a phenotype characterized by a steady increase in disability independent of relapses.^5^ Although most of the disability accrual in the secondary progressive phase is driven by progression independent of relapses, patients may still experience superimposed relapses. The phenotypes RRMS and SPMS do not describe current absence or presence of acute inflammatory disease activity. To ameliorate this challenge, additional descriptors labelled ‘phenotype modifiers’ were introduced to better describe the concurrent disease state of SPMS patients.^3^ Using these modifiers, SPMS patients can be further classified as being ‘active’ or ‘non-active’ according to the occurrence of relapses or MRI activity (gadolinium-enhancing lesions and new or enlarging lesions).

Current disease-modifying therapies (DMTs) approved for multiple sclerosis act on the inflammatory component of the disease and efficiently reduce relapses and signs of radiological disease activity in RRMS.^6^ However, these therapies are insufficient in preventing progression and disability accumulation.^7^ Although there recently has been a movement towards viewing multiple sclerosis as a biological continuum compared with a classification based on clinical features,^8^ regulators still base the approval of new drugs on classical multiple sclerosis phenotypes, and ‘non-active’ SPMS patients remain a group with unmet needs.

A great focus from the pharmaceutical industry has thus been dedicated to developing therapies targeting non-active SPMS patients.^9-11^ In parallel, the scientific community has sought to expand the knowledge regarding SPMS patients.^12^ Multiple sclerosis registries have investigated the frequency, proportion, clinical and demographic characteristics of SPMS patients^13^ as well as predictors of transitioning from RRMS to SPMS.^4,14^ To our knowledge, only two studies have used real-world data to investigate the proportion of active and non-active SPMS patients and differences in demographics and clinical characteristics.^15,16^ They categorized SPMS patients as active or non-active according to clinical and radiological disease activity findings in the year prior to the latest visit or SPMS conversion onset, and analyses were conducted at a cross-sectional level (i.e. keeping the subgroups unchangeable).

However, no studies have investigated how patients potentially fluctuate between an active and a non-active stage in the SPMS phase. Using real-world data from the Danish Multiple Sclerosis Registry (DMSR), we try to fill this gap by exploring patterns and predictors of phenotype transition from RRMS to relapsing or non-relapsing SPMS (nrSPMS; i.e. based on recorded relapse activity) and between relapsing SPMS and nrSPMS stages.

## Materials and methods

### Data source

Clinical and demographic patient data were obtained from the population-based, nationwide DMSR.^17^ The DMSR, established in 1956, contains records of all cases of multiple sclerosis in Denmark since 1948, and data entry on treated patients has been mandatory since DMTs became available in Denmark in 1996. The DMSR covers all 13 multiple sclerosis clinics in Danish public hospitals, being the only units authorized to prescribe and administer DMTs. When a patient enters the registry, data are first entered retrospectively (e.g. date of multiple sclerosis onset, relapses prior to a diagnosis of multiple sclerosis) and then prospectively at every follow-up visit. From the DMSR, we retrieved the following information: sex, age, date of multiple sclerosis onset and symptoms, date of multiple sclerosis diagnosis and disease course, date of SPMS diagnosis (defined as the date the treating physician assigned the SPMS diagnosis), dates of relapses, information about DMT usage (drug type, with initiation and termination dates) and Expanded Disability Status Scale (EDSS)^18^ scores and their dates.

### Study population

**Figure 1:**
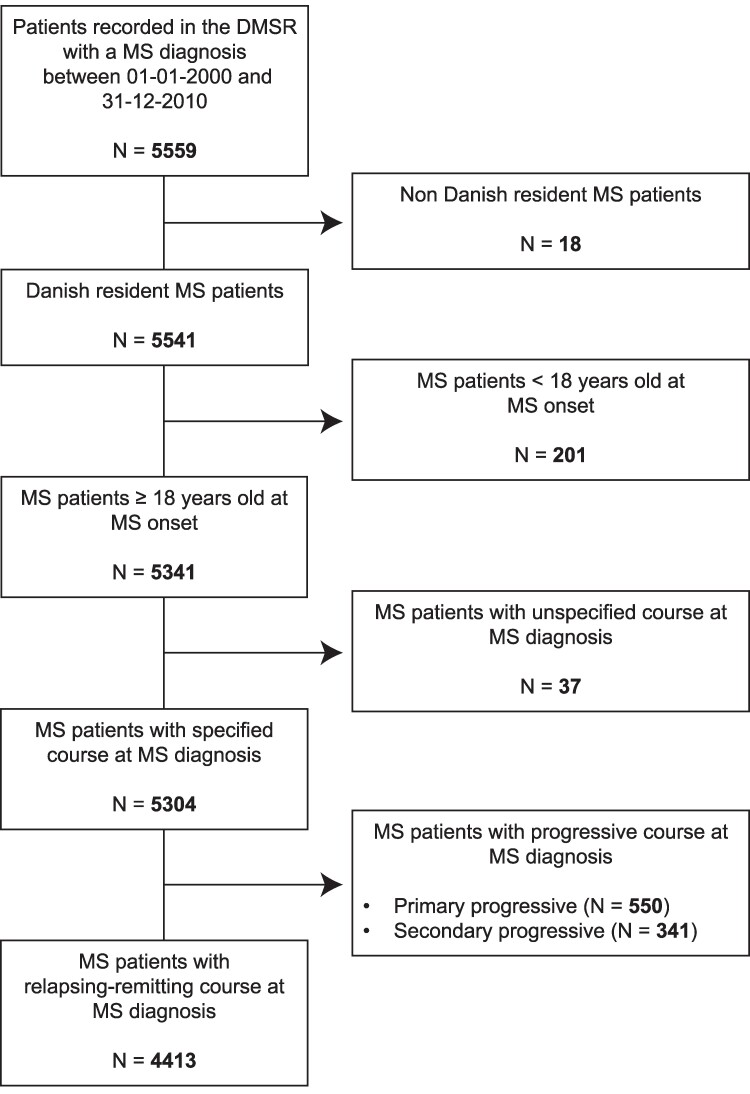
Patients’ disposition flowchart.

We conducted a nationwide, longitudinal cohort study. Inclusion criteria were a diagnosis of multiple sclerosis between 1 January 2000 and 31 December 2010. In addition, a valid Danish Civil Registration Number, ≥18 years of age at onset of multiple sclerosis and a relapsing-remitting course at diagnosis of multiple sclerosis were needed (Fig. 1). Patients were followed from the date of diagnosis until the date of death, emigration, discontinuation from the registry or date of data extraction from the registry (2 September 2022), whichever came first.

### Phenotype identification procedure

**Figure 2:**
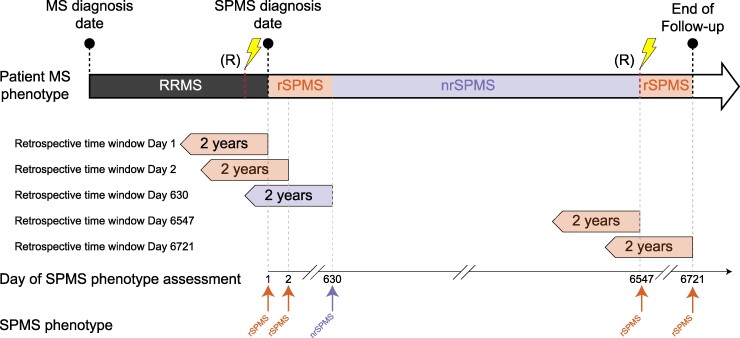
**Example of multiple sclerosis phenotype identification procedure.** The example uses an RRMS patient later diagnosed with SPMS. The multiple sclerosis phenotype is RRMS from the day of multiple sclerosis diagnosis until the day of SPMS diagnosis. At this day (indicated as Day 1 in the ‘Day of SPMS phenotype assessment’ timeline), the patient is deemed rSPMS because a relapse (R) overlaps with the 2-year ‘retrospective time window Day 1’. The patient multiple sclerosis phenotype remains rSPMS until Day 630, when it is deemed nrSPMS, as the application of the retrospective 2-year time window does not overlap with a relapse. At Day 6547, the patient transition again to rSPMS after experiencing a relapse and stays in this category until the end of follow-up (Day 6721).

We assessed the multiple sclerosis phenotype of each patient included in the study at each day of follow-up using the following criteria (example in Fig. 2):

- RRMS: diagnosis of RRMS with no subsequent diagnosis of SPMS.
- nrSPMS: a diagnosis of SPMS according to the treating physician and absence of relapses in the previous 2 years.
- Relapsing SPMS (rSPMS): a diagnosis of SPMS according to the treating physician and at least one relapse in the previous 2 years.

### Statistical analysis

Statistical analyses were performed in SAS version 9.4 (SAS Institute Inc.) and R version 4.1.0.^19^ A *P*-value of <0.05 was considered statistically significant. Patients’ clinical and demographic characteristics at RRMS and SPMS diagnosis dates were summarized using frequency and percentage for categorical variables and mean [standard deviation (SD)] or median and interquartile range (IQR) for continuous variables as appropriate. Point estimates and 95% confidence intervals (CIs) for the annualized relapse rates (ARRs) over the 2 years prior to the date of multiple sclerosis diagnosis were obtained using Poisson regression models that included the logarithm of the follow-up time as an offset term to accommodate patients with <2 years between multiple sclerosis onset and diagnosis.

We used non-parametric Aalen–Johansen estimators to obtain time-specific cumulative probability estimates, adjusted for the competing risk of death, for (i) the transition from RRMS to nrSPMS or rSPMS, (ii) the transition from nrSPMS to the first rSPMS stage for patients classified as nrSPMS at SPMS diagnosis and (iii) the transition from rSPMS to the first nrSPMS stage for patients classified as rSPMS at SPMS diagnosis.

We used multivariable Cox regression models adjusted for age at the onset of multiple sclerosis and sex to assess differences between the rSPMS and nrSPMS sub-phenotypes as classified at the time of SPMS diagnosis. We analysed the rate of reaching EDSS Milestones 3, 4 and 6 prior to SPMS transition as outcomes. In these analyses, patients were followed from the date of multiple sclerosis onset until the outcome or the date of SPMS diagnosis. The proportional hazards assumption of the covariates was assessed by comparing the empirical with the simulated score processes as proposed by Lin *et al.*,^20^ and all covariates fulfilled the proportional hazards assumption. Kaplan–Meier failure curves were used to visualize time-to-event outcomes.

We used a generalizing estimating equation to assess whether patients remaining RRMS during the entire follow-up period, patients classified as nrSPMS at SPMS diagnosis and patients classified as rSPMS at SPMS diagnosis displayed different ARR trajectories during the relapsing-remitting phase of the disease. For every year of the patients’ age, we counted the number of relapses during the relapsing-remitting phase. We defined the relapsing-remitting phase as starting at the clinical onset of multiple sclerosis and lasting to the end of follow-up for patients remaining RRMS and as starting at the onset of multiple sclerosis and lasting until 2 years prior to the date of SPMS diagnosis for nrSPMS and rSPMS patients. For the SPMS patients, we did not include relapses in the 2 years prior to SPMS diagnosis to avoid including relapses that defined the SPMS subgroups. Then, the number of relapses was modelled using a Poisson distribution with a log link function, an offset term given by the logarithm of the follow-up time, and functions for age, sex, multiple sclerosis phenotype and the interaction between multiple sclerosis phenotype and age as the explanatory variables. Given that each patient contributed to the analysis with repeated observations, we included the patient's ID as a subject-effect term and specified an unstructured working correlation matrix. We obtained sex and multiple sclerosis phenotype-specific adjusted ARRs, incidence rate ratios and 95% CIs.

Using a multi-state Markov model from the R package ‘msm’,^21^ we modelled longitudinal transitions of multiple sclerosis phenotype status and the effect of demographic and clinical covariates on the transition intensities (i.e. the instantaneous risk, per time unit, of transitioning from one state to another). In multi-state models, individuals can transition between several well-defined and distinct states. Multi-state models have been used in the context of several progressive diseases, including multiple sclerosis.^22-24^ We defined a four-state Markov model and established constraints on acceptable transitions between states, which included three transient states (RRMS, nrSPMS and rSPMS) and one absorbing state (Death) (Supplementary Fig. 1). The model included both fixed (sex, calendar year of RRMS diagnosis and disease duration at RRMS diagnosis) and time-varying covariates (cumulative years on high-efficacy DMT and moderate-efficacy DMT, age, EDSS score and cumulative number of relapses since RRMS diagnosis). We modelled each transition using a specific set of covariates. We defined high-efficacy DMTs as alemtuzumab, cladribine, daclizumab, fingolimod, natalizumab, ocrelizumab, ofatumumab, ozanimod, rituximab and siponimod. In contrast, we defined moderate-efficacy DMTs as dimethyl fumarate, diroximel fumarate, glatiramer acetate, interferons (β-1a, β-1b and pegylated interferon) and teriflunomide. Model goodness of fit was assessed by visually comparing observed and expected prevalence of multiple sclerosis phenotype states by time (Supplementary Fig. 2). Predicted transition probabilities between multiple sclerosis phenotypes were estimated at 5, 10 and 15 years after diagnosis of RRMS. 95% CIs were obtained from the quantiles of 100 samples drawn from the asymptotic sampling distribution of the estimated log intensities.^25^ Likewise, hazard ratios (HRs) and 95% CIs of the effect of time-fixed and time-varying covariates on specific transition intensities were computed.

## Results

### Characteristics of the study population at inclusion

A total of 4413 patients with an initial RRMS diagnosis were included in the study (Fig. 1). Demographic and clinical characteristics at the time of diagnosis can be seen in Table 1. Most patients were female (70%), with a mean age at onset of multiple sclerosis and diagnosis of RRMS of 34 and 37 years, respectively. The median EDSS at the time of RRMS diagnosis was 2 (IQR: 1.5–3.0), and the mean ARR calculated over the 2 years preceding the diagnosis was 1.72 (95% CI: 1.68–1.77). The most common symptoms at onset were sensory (36.2%), followed by visual (20.1%) and pyramidal tract (13.6%). The cohort was followed for a median of 16.2 years (IQR: 13.5–19.2).

**Table 1:** Demographic and clinical characteristics of included patients

	RRMS (*N* = 4413)
Sex, female, *n* (%)	3087 (70.0)
Age at onset of multiple sclerosis, years, mean ± SD	34.1 ± 9.2
Age at RRMS diagnosis, years, mean ± SD	37.5 ± 9.6
Disease duration (since multiple sclerosis onset), years, median (IQR)	1.0 (0.1–4.4)
EDSS at RRMS diagnosis, median (IQR)^a^	2.0 (1.5–3.0)^b^
Relapses 2 years prior to RRMS diagnosis, *n* (%)	
0 relapse	201 (4.6)
1 relapse	1342 (30.4)
>1 relapses	2870 (65.0)
ARR 2 years prior to RRMS diagnosis, mean (95% CI)^c^	1.72 (1.68–1.77)
Type of symptoms at multiple sclerosis onset, *n* (%)	
Sensory	1598 (36.2)
Opticus	885 (20.1)
Pyramidal	600 (13.6)
Multi-focal	435 (9.9)
Brainstem	298 (6.8)
Cerebellar	217 (4.9)
Sphincter	58 (1.3)
Other	157 (3.6)
Unknown	165 (3.7)
Follow-up duration, years, median (IQR)	16.2 (13.5–19.2)

^a^EDSS score closer to the multiple sclerosis diagnosis date within a ±1-year time window.

^b^Calculated on 3083 patients with an available EDSS score.

^c^Values obtained using a Poisson regression model with robust error variance.

### Characteristics of relapsing and non-relapsing secondary progressive multiple sclerosis patients at secondary progressive multiple sclerosis diagnosis

**Figure 3:**
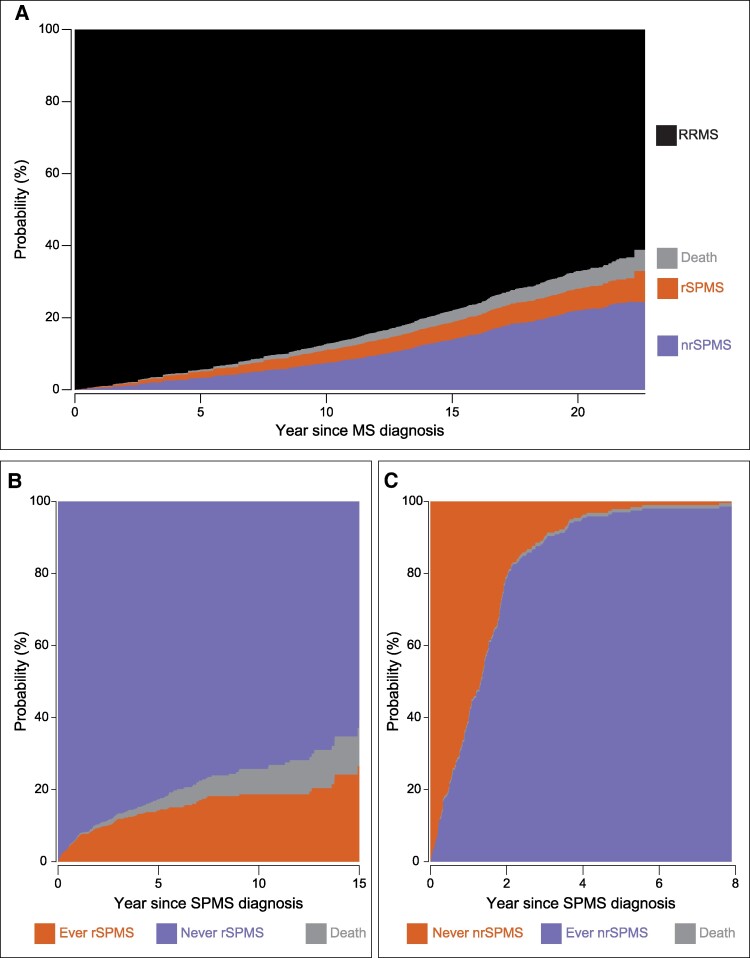
**Aalen–Johansen cumulative incidence functions estimate stacked plots of multiple sclerosis phenotype transitions in the selected population.** (**A**) Cumulative risk of receiving an SPMS diagnosis with a non-relapsing (violet) or relapsing (orange) course, staying event-free (black) or dying (grey) for the initial RRMS population (*n* = 4413) over a maximum follow-up time of 22.8 years. (**B**) Cumulative risk of ever transitioning to a rSPMS phase, dying or remaining relapse-free for the nrSPMS population at SPMS diagnosis (*n* = 962) over a maximum follow-up time of 15 years since SPMS diagnosis. (**C**) Cumulative risk of ever transitioning to an nrSPMS phase, dying or never entering a nrSPMS for the rSPMS population at SPMS diagnosis (*n* = 233) over a maximum follow-up time of 7.8 years since SPMS diagnosis. In all panels, the distances between two adjacent curves represent the cumulative probabilities, expressed as percentages, of the different events.

Of the 4413 patients constituting our initial cohort, 962 received a diagnosis of SPMS over a maximum follow-up of 22.8 years (cumulative probability at the end of follow-up: 32.9%, Fig. 3A). Using our operational definition (Fig. 2), 233 of these patients could be classified as rSPMS at the date of SPMS diagnosis, whereas 729 were classified as nrSPMS. By the end of follow-up, the cumulative probabilities of receiving an SPMS diagnosis and being nrSPMS or rSPMS were 24.3 and 8.6%, respectively (Fig. 3A). Demographic and clinical characteristics of these two subgroups of SPMS patients can be seen in Table 2, along with characteristics of patients remaining RRMS.

**Table 2:** Demographic and clinical characteristics of patients by multiple sclerosis phenotype

	RRMS (*N* = 3451)	rSPMS (*N* = 233)	nrSPMS (*N* = 729)
Sex, female, *n* (%)	2491 (72.2)	146 (62.7)	450 (61.7)
Age at onset of multiple sclerosis, years, mean ± SD	33.4 ± 9.0	35.2 ± 9.0	37.1 ± 9.7
Age at RRMS diagnosis, years, mean ± SD	36.4 ± 9.3	39.8 ± 9.3	42.0 ± 9.6
Age at SPMS diagnosis, years, mean ± SD		47.9 ± 9.0	52.5 ± 9.7
Time between multiple sclerosis onset and RRMS diagnosis, years, median (IQR)	1.0 (0.1–4.0)	2.0 (0.7–5.9)	2.0 (0.6–7.0)
Time between multiple sclerosis onset and SPMS diagnosis, years, median (IQR)		12.0 (7.4–17.9)	14.5 (10.4–19.0)
EDSS at RRMS diagnosis, median (IQR)^a^	2.0 (1.0–2.5)^b^	2.5 (2.0–3.5)^b^	2.5 (2.0–3.5)^b^
Last EDSS prior to SPMS diagnosis, median (IQR)		5.0 (3.5–6.0)^c^	4.5 (3.0–6.0)^c^
Relapses 2 years prior to RRMS diagnosis, *n* (%)			
0 relapse	144 (4.2)	7 (3.0)	50 (6.9)
1 relapse	1070 (31.0)	53 (22.7)	219 (30.0)
>1 relapses	2237 (64.8)	173 (74.2)	460 (63.1)
ARR 2 years prior to RRMS diagnosis, mean (95% CI)^d^	1.80 (1.74–1.85)	1.68 (1.51–1.87)	1.43 (1.34–1.52)
Follow-up duration, years, median (IQR)	16.2 (13.2–19.2)	17.2 (14.2–20.2)	17.5 (15.2–20.2)

Values are reported for patients who remained RRMS for the whole follow-up and for patients classified as rSPMS and nrSPMS at the time of SPMS diagnosis.

^a^EDSS score closer to the multiple sclerosis diagnosis date within a ±1-year time window.

^b^Calculated on patients with an available EDSS score (2432 RRMS; 178 rSPMS and 473 nrSPMS).

^c^Calculated on patients with an available EDSS score (223 rSPMS and 678 nrSPMS).

^d^Values obtained using separate Poisson regression models with robust error variance.

Females accounted for 61.7, 62.7 and 72.2% among the nrSPMS, rSPMS and RRMS patients, respectively. The mean age in the rSPMS, nrSPMS and RRMS groups was 35.2 versus 37.1 versus 33.4 years at multiple sclerosis onset and 39.8 versus 42.0 versus 36.4 years at RRMS diagnosis. At SPMS diagnosis, the mean age in the rSPMS and nrSPMS groups was 47.9 versus 52.5 years, while the median EDSS was 5.0 (IQR: 3.5–6.0) versus 4.5 (3.0–6.0). Among rSPMS, nrSPMS and RRMS, the ARR 2 years prior to RRMS diagnosis was 1.68 (95% CI: 1.51–1.87), 1.43 (95% CI: 1.34–1.52) and 1.80 (95% CI: 1.74–1.85).

### Time to reach disability milestones and annualized relapse rate among patients later diagnosed with secondary progressive multiple sclerosis

During the relapsing-remitting phase of the disease patients later diagnosed with rSPMS reached EDSS 3 [adjusted HR and 95% CI: 1.427 (1.210–1.676)], EDSS 4 [1.557 (1.297–1.859)] and EDSS 6 [1.857 (1.463–2.342)] faster than patients diagnosed with nrSPMS (Supplementary Fig. 3 and Table 1).

Furthermore, rSPMS had a higher ARR in the relapsing-remitting phase of the disease compared with both nrSPMS and patients who remained RRMS [adjusted ARR (95% CI): rSPMS = 0.442 (0.376–0.508); nrSPMS = 0.197 (0.176–0.219) and RRMS = 0.131 (0.125–0.138); Supplementary Table 2]. In addition, predicted ARR by age and stratified by sex appeared to follow a similar trend among nrSPMS and RRMS, while rSPMS appeared to have higher an ARR at each specific age (Supplementary Fig. 4).

### Patterns of transition between relapsing and non-relapsing secondary progressive multiple sclerosis phases

Of the 729 patients classified as nrSPMS at SPMS diagnosis, 620 remained nrSPMS, whereas 109 had one or more relapses. The cumulative probability for an initially diagnosed nrSPMS patient to never relapse was 60.6% after ∼15 years since SPMS diagnosis, whereas the cumulative probability to ever relapsing was 26.5% (Fig. 3B). Of the 233 patients classified as rSPMS at SPMS diagnosis, 10 never transitioned to a nrSPMS stage, whereas 223 experienced at least 2 consecutive years with no relapses. The cumulative probability for an initially diagnosed rSPMS patient to enter an nrSPMS phase was 99.1% after ∼8 years since SPMS diagnosis, whereas the cumulative probability of never experiencing a nrSPMS phase was 0.5% (Fig. 3C). Furthermore, 152 of the 223 initially diagnosed rSPMS patients who ever experienced an nrSPMS stage remained relapse-free for the remaining of the follow-up. A full breakdown of the SPMS subtype transition patterns identified in the SPMS population, as well as number of patients experiencing each type of transition pattern, can be seen in Supplementary Table 3.

### Transition probabilities and predictors of phenotypic transition

Covariates-adjusted cumulative transition probabilities obtained from the Markov multi-state model revealed that RRMS patients had a 94.6% (95% CI: 94.1–95.0) probability of remaining RRMS 5 years after RRMS diagnosis. This probability decreased to 89.4% (95% CI: 88.6–90.2) and 84.6% (95% CI: 83.3–85.7) after 10 and 15 years, respectively. Moreover, nrSPMS patients had a probability of transitioning to rSPMS of 13.4% (11.0–16.1) after 5 years, 13.5% (10.9–16.3) after 10 years and 13.2% (10.7–16.0) after 15 years from RRMS diagnosis. Additionally, rSPMS patients had a probability of moving to an nrSPMS phase of 82.7% (95% CI: 79.1–85.5), 83.1% (95% CI: 78.3–86.0) and 81.6% (95% CI: 75.4–85.1) 5, 10 and 15 years after RRMS diagnosis, respectively (Table 3).

**Table 3:** Covariates-adjusted cumulative transition probabilities across multiple sclerosis phenotypes

	RRMS	nrSPMS	rSPMS
5-year transition probabilities, % (95% CIs)			
RRMS	94.6 (94.1–95.0)	3.9 (3.6–4.3)	0.9 (0.7–1.0)
nrSPMS		84.6 (81.1–87.1)	13.4 (11.0–16.1)
rSPMS		82.7 (79.1–85.5)	15.9 (13.0–19.3)
10-year transition probabilities, % (95% CIs)			
RRMS	89.4 (88.6–90.2)	7.7 (7.0–8.4)	1.5 (1.2–1.8)
nrSPMS		82.7 (77.3–85.8)	13.5 (10.9–16.3)
rSPMS		83.1 (78.3–86.0)	13.6 (11.1–16.5)
15-year transition probabilities, % (95% CIs)			
RRMS	84.6 (83.3–85.7)	11.2 (10.2–12.2)	2.0 (1.7–2.4)
nrSPMS		81.1 (74.3–84.8)	13.2 (10.7–16.0)
rSPMS		81.6 (75.4–85.1)	13.3 (10.8–16.1)

Estimated 5-, 10- and 15-year covariates-adjusted cumulative transition probabilities from RRMS diagnosis within and to each SPMS phenotype. Transition probabilities between the same phenotypes should be interpreted as the probability that a patient does not change its classification at the specified time point (e.g. a patient classified as RRMS at Year 0 has a 94.6% chance to be still RRMS after 5 years).

Results from the multi-state Markov model (Table 4) showed that female RRMS patients had a lower rate of transitioning to both nrSPMS [HR (95% CI): 0.735 [0.619–0.874]) and rSPMS [HR (95% CI): 0.719 (0.548–0.945)] compared with males. However, once in an SPMS state, sex did not significantly influence the transitions from nrSPMS to rSPMS and vice versa. Older age increased the rate of transitioning from RRMS to nrSPMS [HR (95% CI): 1.044 (1.035–1.053)] but not to rSPMS [HR (95% CI): 1.009 (0.996–1.023)]. In addition, when in an nrSPMS stage, older age decreased the rate of transitioning to a rSPMS state [HR (95% CI): 0.956 (0.942–0.971)]. RRMS patients who spent more years on high and moderate DMTs also had a higher rate of transitioning to nrSPMS [HR_HeDMT_ (95% CI): 1.095 (1.051–1.141) and HR_MeDMT_ (95% CI): 1.073 (1.051–1.095)], but time on DMTs did not affect any other transitions. Every 1-unit increase in the EDSS score, which was allowed to vary over time, resulted in a 52% [HR (95% CI): 1.522 (1.458–1.590)] and 81% [HR (95% CI): 1.810 (1.686–1.944)] higher rate of transitioning from RRMS to nrSPMS and rSPMS, respectively. Also, for every additional relapse, the rate of transitioning from RRMS to rSPMS would increase by 8% [HR (95% CI): 1.077 (1.051–1.103)], and the rate of transitioning to nrSPMS would decrease by 14% [HR (95% CI): 0.863 (0.823–0.906)].

**Table 4:** Covariates effect on multiple sclerosis phenotype transition probabilities

From state →	RRMS	RRMS	nrSPMS	rSPMS
To state →	nrSPMS	rSPMS	rSPMS	nrSPMS
	HR (95% CI)	HR (95% CI)	HR (95% CI)	HR (95% CI)
Sex (reference = male)^a^	**0.735** (**0.619–0.874)**	**0.719** (**0.548–0.945)**	1.239 (0.923–1.664)	0.881 (0.724–1.072)
Age^b^	**1.044** (**1.035–1.053)**	1.009 (0.996–1.033)	**0.956** (**0.942–0.971)**	1.002 (0.991–1.012)
Cumulative time on HeDMT since RRMS diagnosis^b^	**1.095** (**1.051–1.141)**	0.989 (0.932–1.050)	1.040 (0.990–1.092)	0.981 (0.945–1.017)
Cumulative time on MeDMT since RRMS diagnosis^b^	**1.073** (**1.051–1.095)**	0.992 (0.953–1.033)	0.976 (0.942–1.011)	0.992 (0.966–1.020)
EDSS^b^	**1.522** (**1.458–1.590)**	**1.810** (**1.686–1.944)**	0.972 (0.903–1.046)	1.024 (0.964–1.087)
Calendar year of RRMS diagnosis^b^	1.003 (0.976–1.030)	1.033 (0.990–1.078)	^c^	^c^
Cumulative number of relapses since RRMS diagnosis^b^	**0.863** (**0.823–0.906)**	**1.077** (**1.051–1.103)**	^c^	^c^
Disease duration^b^	1.017 (1.004–1.030)	1.020 (0.998–1.043)	^c^	^c^

Effect of time-fixed and time-varying covariates on the HR of transitioning between multiple sclerosis phenotypes over the follow-up period. Bold indicates statistically significant values (*P* < 0.05).

^a^Categorical covariate.

^b^Continuous covariate.

^c^Covariate not included in the model for the specific transition.

## Discussion

In this study, we characterized how patients with RRMS transition to rSPMS or nrSPMS and subsequent transitions between these phases of the SPMS course. Furthermore, we identified significant demographic and clinical predictors of phenotype transition.

The probability for RRMS patients to be diagnosed with nrSPMS or rSPMS in our cohort with a median follow-up time of 16.2 years was generally low (11.2 and 2.0% after 15 years of follow-up, respectively), and overall, the cumulative probability of receiving a clinical diagnosis of SPMS by the end of the follow-up was 32.9%. This probability is smaller than that reported in other natural history cohorts.^4,26^ One possible explanation for this discrepancy might be the relatively short follow-up in this study. With a longer follow-up, we might have found a higher proportion of patients receiving a diagnosis of SPMS. However, another explanation could be linked to the challenges experienced by physicians when diagnosing SPMS. Challenges include the absence of a distinct biomarker, the limited availability of DMTs for SPMS, how patients and society view SPMS, and the time required to confirm progression.^27,28^ The interplay of these factors often leads to a delay and under-diagnosis of SPMS.^29^ Therefore, the low SPMS incidence observed in our study is likely to be an underestimation of the ‘true’ incidence of SPMS patients. The recent application of ‘SPMS objective classifiers’ to large real-world cohorts of RRMS patients indeed suggests that the proportion of SPMS patients might be higher and that the time to receive an SPMS diagnosis could be shorter.^13,27,30,31^

In our cohort, most patients who transitioned to SPMS were classified as nrSPMS at the time of SPMS diagnosis (629 of 962). These patients reached disability milestones more slower and had a lower ARR during the RRMS phase compared with patients initially classified as rSPMS. The challenges in diagnosing SPMS in patients with RRMS, besides potentially affecting the overall prevalence in our population, also appear to influence the likelihood of being classified as rSPMS or nrSPMS. Also, the predictors significantly associated with the probability of being classified as nrSPMS coincide with those most commonly found associated with a lower risk of relapses. Male sex, older age and extended time on DMTs were significant risk factors for the transition from RRMS to nrSPMS. This is in line with findings from other studies indicating that male sex is a risk factor for SPMS transition,^32,33^ as well as studies indicating that males experience fewer relapses than females.^34^ Furthermore, relapses tend to fade with age,^35^ and an interaction between sex and age has been shown in several studies, with sex differences in relapses usually disappearing after the age of 50, coinciding with females entering menopause.^34,36^ Finally, the use of DMTs, and particularly the use of high-efficacy drugs, can potently suppress relapse occurrence. As DMTs are primarily effective in suppressing inflammatory activity, this could potentially unmask underlying progression.^37^

A higher EDSS at the time of transition also influenced transition probabilities from RRMS to both nrSPMS and rSPMS. This may indicate that physicians appear more prone to assign an SPMS diagnosis when the EDSS score has reached a certain threshold. Similarly, objective SPMS classifiers developed in recent years, using clinical SPMS assignment as a gold standard, require a high or even a minimal EDSS threshold to be reached to be classified as SPMS.^30,31^ While a high EDSS score is expected to be associated with being classified as SPMS, Lublin *et al*.^3,38^ did not establish a minimal EDSS threshold that the patient must reach to be diagnosed as SPMS.

While the first transition to either nrSPMS or rSPMS appears to be affected by the inherent biases affecting SPMS assignment in the first place, subsequent transitions (e.g. from nrSPMS to rSPMS and vice versa) seem to be freed from these biases. Sex, time on DMTs and EDSS were not associated with the risk of further transitions after reaching rSPMS or nrSPMS. The only predictor associated with subsequent transitions was the age for the transition from nrSPMS to rSPMS. Specifically, older age would reduce the risk of entering a relapsing phase during the secondary progressive period. As previously mentioned, this is in line with other studies reporting that relapse activity diminishes with age.^35^

Recently, a view of multiple sclerosis as a biological continuum instead of distinct clinical phenotypes has gained attention. It is argued that multiple sclerosis patients share the same types of pathology features, although the extent can vary and that these differences can be influenced by sex, age, social and environmental exposures, genetic factors and disease duration.^8^ This view is not in contrast to our study since we found sex and age to be risk factors for SPMS and age to be a risk factor for transitioning between SPMS states.

Our study has some key limitations. First, as argued, the use of the date of SPMS diagnosis and the length of follow-up might have led to an underestimation of the overall proportion of patients identified as SPMS. As previously argued, restraint in diagnosing SPMS due to a limited number of DMTs available for SPMS could also have affected the proportion of patients diagnosed with SPMS. Second, we did not use MRI activity in our operational definition of relapsing SPMS and nrSPMS. This is because the DMSR only started to systematically collect MRI information in 2015. As disease activity can be measured as either relapse occurrence or active MRI lesions, the phenotype descriptor definition proposed by Lublin *et al*.^38^ would still allow us to define the nrSPMS group as ‘non-active’ and the rSPMS as ‘active’. However, we preferred to define our groups as relapsing and non-relapsing to better clarify that we only used relapses, thereby preventing additional confusion regarding how activity is measured. While the availability of MRI information might have revealed that some of the patients classified as nrSPMS would actually be classified as active SPMS if imaging data were used in the operational definition of activity, we argue that MRI lesions and relapse activity are closely linked, with relapses representing clinical disease activity readily available to judge for all physicians. Furthermore, although the suggested time window for activity measurement is recommended to be 1 year,^3^ we used a 2-year time window as clinical visits can be more than 1 year apart, particularly for patients who are not treated with DMTs.

Our study strengths were access to nationwide data coverage on patients with multiple sclerosis and the use of a large, representative cohort of RRMS patients (*n* = 4413) followed for a median of 16.2 years since diagnosis of multiple sclerosis.

## Conclusion

Nine hundred sixty-two of 4413 RRMS patients received an SPMS diagnosis by the end of follow-up (∼22 years). The majority of these (729) were initially in an nrSPMS state, and 620 never experienced relapses over the remaining follow-up. Our study suggests that the transition from RRMS to relapsing SPMS or nrSPMS highly depends on well-known factors affecting SPMS diagnosis. Subsequent transitions during the secondary progressive stage, instead, display a pattern consistent with the expected effect of age on important clinical parameters. These results can have implications in terms of characterizing patients, who can be the target of new therapies aimed specifically at the non-active phase of the secondary progressive stage of multiple sclerosis.

## Supplementary Material

fcae422_Supplementary_Data

## Data Availability

The data and the analysis scripts that support the findings of this study cannot be shared publicly due to data protection regulation. Anonymized data and scripts will be shared, upon reasonable request, with authorized researchers after application to the Danish Health Data Authority and the board of the Danish Multiple Sclerosis Registry.
