# Supplementary material for: Patterns and predictors of multiple sclerosis phenotype transition
Source: Brain Commun. 2024 Nov 23;6(6):fcae422. doi: 10.1093/braincomms/fcae422 (PMC11660925; doi:10.1093/braincomms/fcae422)
Supplement: fcae422_Supplementary_Data [file fcae422_supplementary_data.pdf]

Supplementary Materials

Supplementary Figure 1. Multiple sclerosis phenotype transition scheme employed in our study. States (boxes) and allowed transitions (arrows) are shown. Death is defined as an absorbing state. RRMS = relapsing-remitting Multiple Sclerosis; nrSPMS = non-relapsing Secondary Progressive Multiple Sclerosis; rSPMS = relapsing Secondary Progressive Multiple Sclerosis.

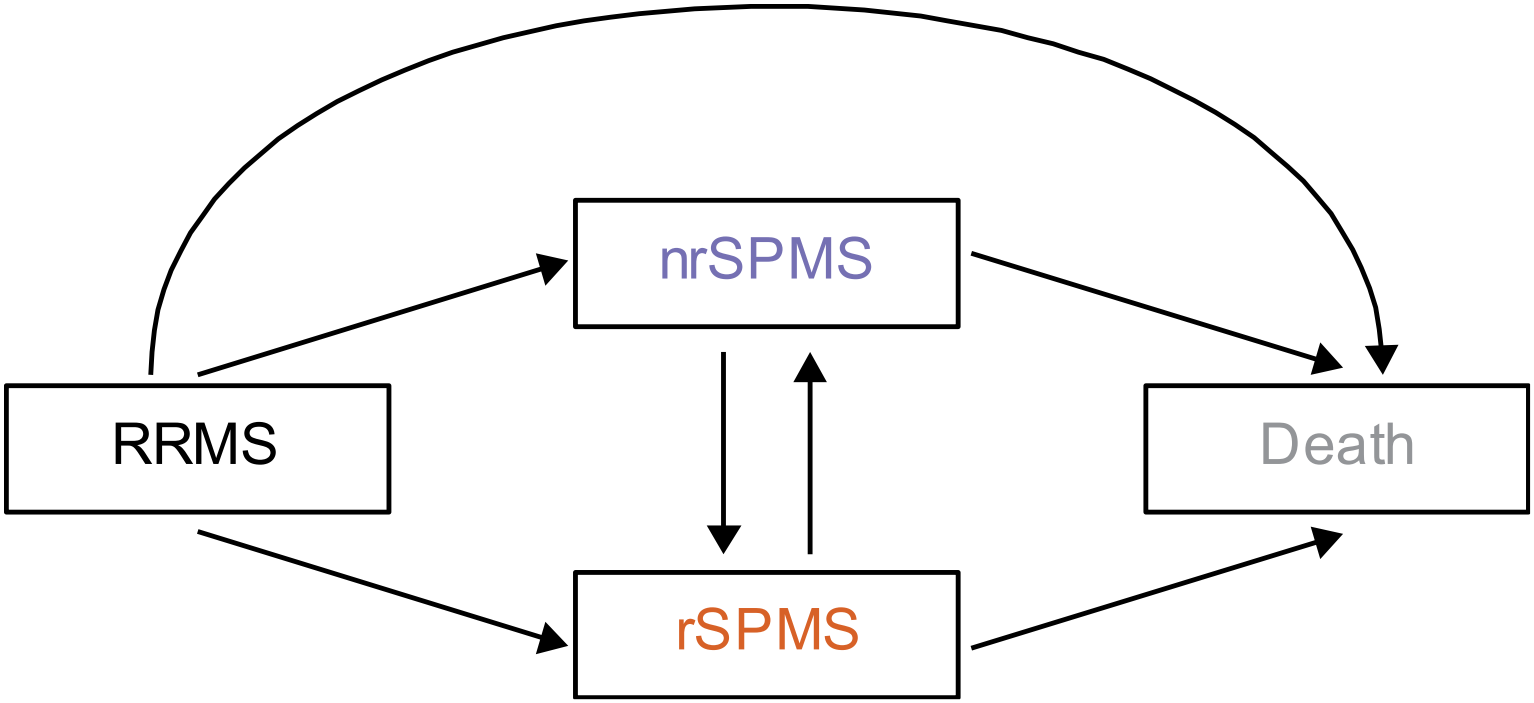

Supplementary Figure 2. Assessment of goodness of fit for the multi-state Markov model comparing observed and expected prevalence of multiple sclerosis phenotype states by time. RRMS = relapsing-remitting Multiple Sclerosis; nrSPMS = non-relapsing Secondary Progressive Multiple Sclerosis; rSPMS = relapsing Secondary Progressive Multiple Sclerosis.

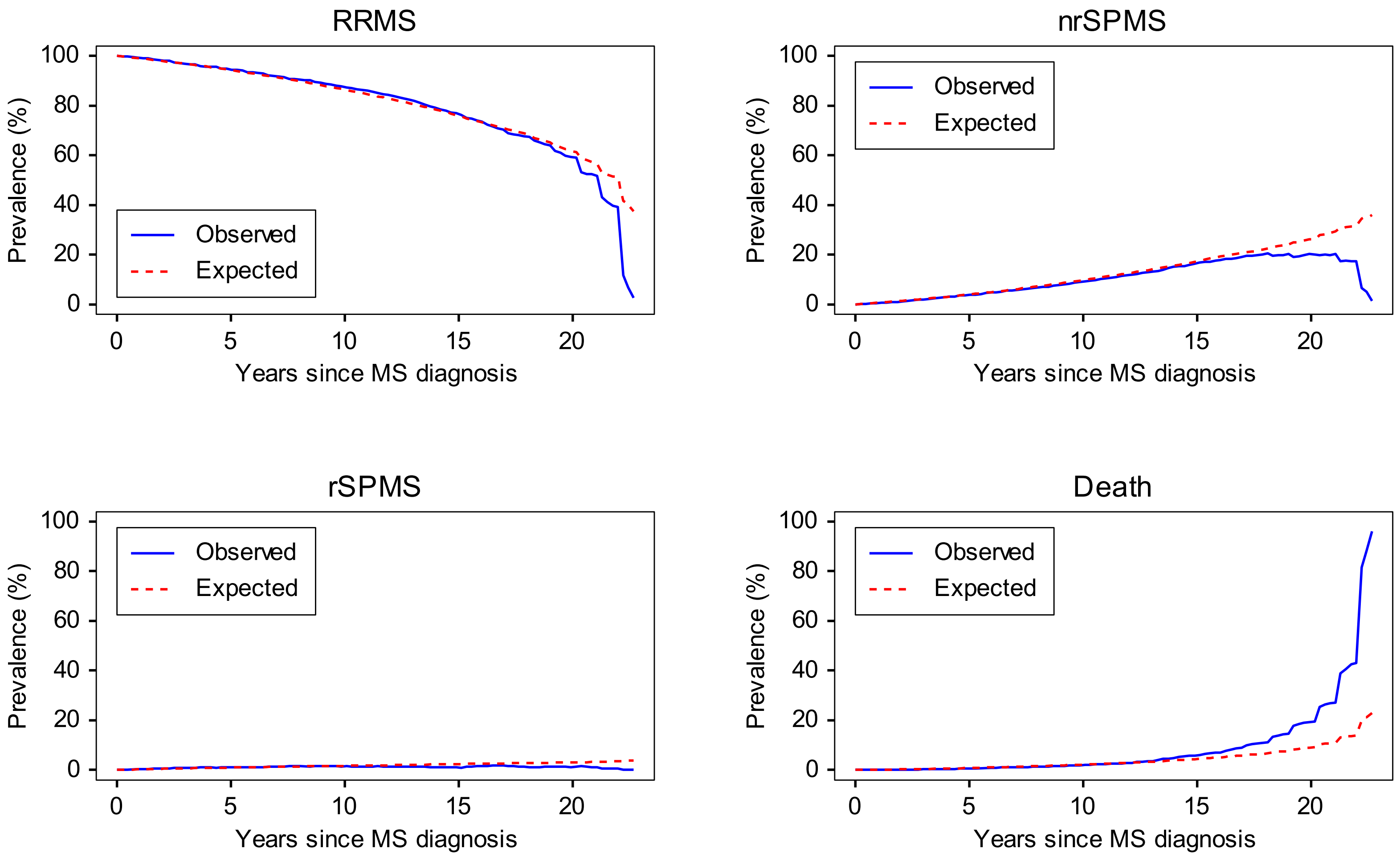

Supplementary Figure 3. Time to reach first Expanded Disability Status Scale (EDSS) milestones since multiple sclerosis diagnosis for patients categorized as non-relapsing secondary progressive multiple sclerosis (nrSPMS, violet) and relapsing secondary progressive multiple sclerosis (rSPMS, orange) at the date of SPMS diagnosis. Time to reach first EDSS 3 **(A)**, 4 **(B)** and 6 **(C)** are displayed using Kaplan-Meier curves. For these analyses, patients were followed until the date of SPMS diagnosis. In all panels, number of patients at risk are reported below the x-axis. Within each figure panel, the frequency and proportion of patients reaching the EDSS milestone and the median time and 95% CIs to reach the EDSS milestone for each SPMS subtype are reported.

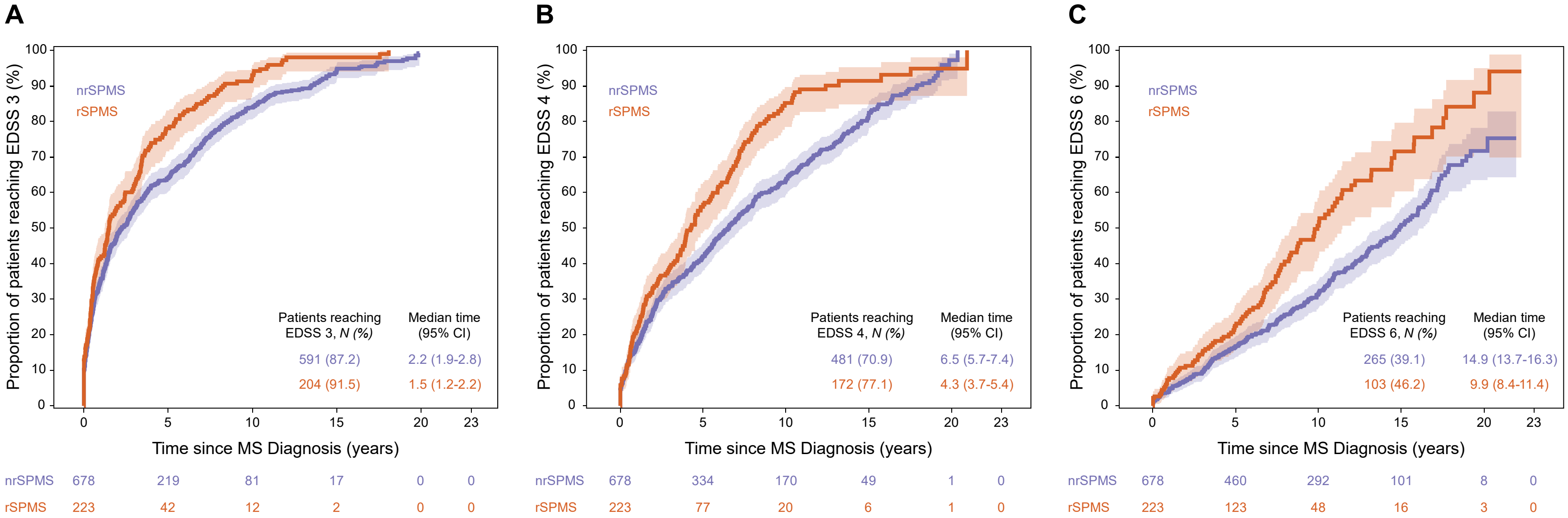

Supplementary Table 1. Multivariable Cox regression analyses of time to reach first EDSS 3, 4 and 6 for patients categorized as non-relapsing secondary progressive multiple sclerosis (nrSPMS) and relapsing secondary progressive multiple sclerosis (rSPMS) at the date of SPMS diagnosis. For these analyses, patients were followed until the date of SPMS diagnosis. All analyses were adjusted for age at multiple sclerosis diagnosis and sex.

Variable	Time to EDSS 3			Time to EDSS 4			Time to EDSS 6		
	HR	95% CI	p-value	HR	95% CI	p-value	HR	95% CI	p-value
rSPMS, *reference = nrSPMS*	1.427	1.210 – 1.676	< 0.0001	1.557	1.297 – 1.859	< 0.0001	1.857	1.463 – 2.342	< 0.0001
Female, *reference = Male*	0.757	0.656 – 0.874	0.0001	0.694	0.593 – 0.813	< 0.0001	0.739	0.601 – 0.910	0.0042
Age at multiple sclerosis diagnosis	1.009	1.002 – 1.016	0.0173	1.008	1.000 – 1.017	0.0525	1.022	1.010 – 1.033	0.0002

EDSS = Expanded Disability Status Scale; nrSPMS = non-relapsing Secondary Progressive Multiple Sclerosis; rSPMS = relapsing Secondary Progressive Multiple Sclerosis.

Supplementary Table 2. Adjusted annualized relapse rates (ARRs) and incidence rate ratios (IRRs) for the three multiple sclerosis phenotypes. Patients in the progressive phenotype categories were categorized with the secondary progressive multiple sclerosis (SPMS) subtype defined at the day of SPMS diagnosis [relapsing SPMS (rSPMS) or non-relapsing SPMS (nrSPMS)] and followed until 2 years prior to the date of SPMS diagnosis. Patients who remained relapsing-remitting (RRMS) were followed until the end of follow-up. Adjusted point estimates for the three multiple sclerosis phenotype categories were obtained from a model including age, sex, multiple sclerosis phenotype category and the interaction between age and multiple sclerosis phenotype category.

	Adjusted ARR	95% CI	Adjusted IRR	95% CI
RRMS	0.131	0.125 – 0.138	reference	reference
nrSPMS	0.197	0.176 – 0.219	1.507	1.335 – 1.702
rSPMS	0.442	0.376 – 0.508	3.377	2.885 – 3.953

RRMS = relapsing-remitting Multiple Sclerosis; nrSPMS = non-relapsing Secondary Progressive Multiple Sclerosis; rSPMS = relapsing Secondary Progressive Multiple Sclerosis; ARR = Annualized Relapse Rate; CI = Confidence Interval; IRR = Incidence Rate Ratio.

Supplementary Figure 4. Observed (filled circles) and predicted (solid lines) annualized relapse rates (ARRs) at every age for the three multiple sclerosis phenotypes, stratified by male **(A)** and female **(B)** patients. Patients in the progressive phenotype categories were categorized with the secondary progressive multiple sclerosis (SPMS) subtype defined at the day of SPMS diagnosis [relapsing SPMS (rSPMS, N = 233) or non-relapsing SPMS (nrSPMS, N = 729)] and followed until 2 years prior to the date of SPMS diagnosis. Patients who remained relapsing-remitting (RRMS, N = 3451) were followed until the end of follow-up. ARRs were calculated at every age using the number of relapses experienced in the previous year divided by the follow-up time in the previous year. Predicted values were obtained from a model including age, sex, multiple sclerosis phenotype category and the interaction between age and multiple sclerosis phenotype category. Bands surrounding the predicted values indicates 95% confidence intervals of the predictions.

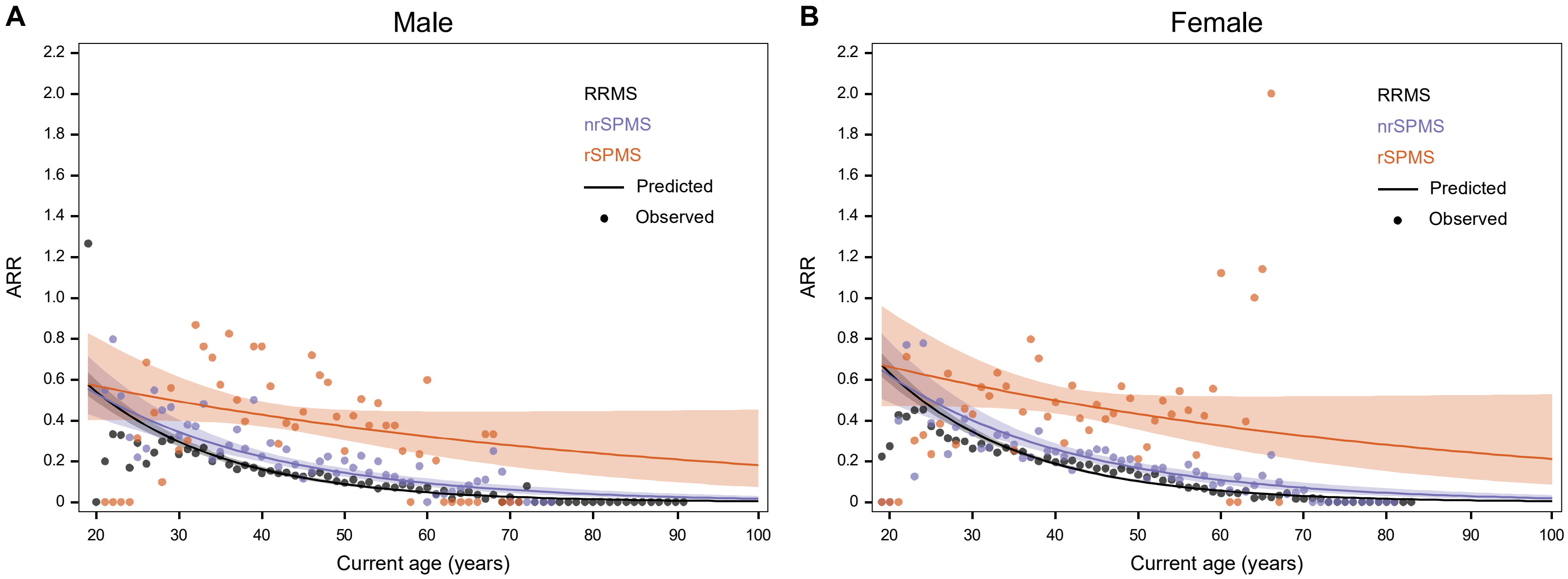

Supplementary Table 3. SPMS subtypes transition patterns identified in the study population. The table reports the number of patients (column "N patients") experiencing a specific SPMS subtype transition pattern as identified after applying the rSPMS and nrSPMS operational definition described in Figure 2 (column "SPMS subtype transition pattern during follow-up). The table also reports the SPMS subtype classification identified at SPMS diagnosis date and at the end of follow-up, the number of transition events that each transition pattern contributes, and the total number of nrSPMS → rSPMS and rSPMS → nrSPMS transition events the pattern displays. As patients can contribute to multiple transitions, between parentheses we report the number of patients contributing to the pattern.

SPMS subtype (SPMS diagnosis date)	SPMS subtype (End of follow-up)	SPMS subtype transition pattern during follow-up	N patients	Number of transition events per pattern	Number of nrSPMS → rSPMS transition events (N patients)	Number of rSPMS → nrSPMS transition events (N patients)
nrSPMS	nrSPMS	No transition to rSPMS subtype during follow-up	620	0	-	-
nrSPMS	nrSPMS	nrSPMS → rSPMS → nrSPMS	72	2	72 (72)	72 (72)
nrSPMS	nrSPMS	nrSPMS → rSPMS → nrSPMS → rSPMS → nrSPMS	17	4	34 (17)	34 (17)
nrSPMS	nrSPMS	nrSPMS → rSPMS → nrSPMS → rSPMS → nrSPMS → rSPMS → nrSPMS	3	6	9 (3)	9 (3)
nrSPMS	rSPMS	nrSPMS → rSPMS	14	1	14 (14)	-
nrSPMS	rSPMS	nrSPMS → rSPMS → nrSPMS → rSPMS	3	3	6 (3)	3 (3)
rSPMS	rSPMS	No transition to nrSPMS subtype during follow-up	10	0	-	-
rSPMS	rSPMS	rSPMS → nrSPMS → rSPMS	9	2	9 (9)	9 (9)
rSPMS	rSPMS	rSPMS → nrSPMS → rSPMS → nrSPMS → rSPMS	3	4	6 (3)	6 (3)
rSPMS	rSPMS	rSPMS → nrSPMS → rSPMS → nrSPMS → rSPMS → nrSPMS → rSPMS	2	6	6 (2)	6 (2)
rSPMS	nrSPMS	rSPMS → nrSPMS	152	1	-	152 (152)
rSPMS	nrSPMS	rSPMS → nrSPMS → rSPMS → nrSPMS	49	3	49 (49)	98 (49)
rSPMS	nrSPMS	rSPMS → nrSPMS → rSPMS → nrSPMS → rSPMS → nrSPMS	7	5	14 (7)	21 (7)
rSPMS	nrSPMS	rSPMS → nrSPMS → rSPMS → nrSPMS → rSPMS → nrSPMS → rSPMS → nrSPMS	1	7	3 (1)	4 (1)
		Total	962	-	222 (180)	414 (318)
